# Lessons from two high CO_2_ worlds – future oceans and intensive aquaculture

**DOI:** 10.1111/gcb.13515

**Published:** 2016-10-20

**Authors:** Robert P. Ellis, Mauricio A. Urbina, Rod W. Wilson

**Affiliations:** ^1^ BiosciencesUniversity of ExeterGeoffrey Pope BuildingStocker RoadEX4 4QDExeterUK; ^2^ Departamento de ZoologíaFacultad de Ciencias Naturales y OceanográficasUniversidad de ConcepciónCasilla 160‐CConcepción4070386Chile

**Keywords:** aquatic carbonation, carbon dioxide, climate change, food security, ocean acidification, recirculating aquaculture system

## Abstract

Exponentially rising CO_2_ (currently ~400 μatm) is driving climate change and causing acidification of both marine and freshwater environments. Physiologists have long known that CO_2_ directly affects acid–base and ion regulation, respiratory function and aerobic performance in aquatic animals. More recently, many studies have demonstrated that elevated CO_2_ projected for end of this century (e.g. 800–1000 μatm) can also impact physiology, and have substantial effects on behaviours linked to sensory stimuli (smell, hearing and vision) both having negative implications for fitness and survival. In contrast, the aquaculture industry was farming aquatic animals at CO_2_ levels that far exceed end‐of‐century climate change projections (sometimes >10 000 μatm) long before the term ‘ocean acidification’ was coined, with limited detrimental effects reported. It is therefore vital to understand the reasons behind this apparent discrepancy. Potential explanations include 1) the use of ‘control’ CO_2_ levels in aquaculture studies that go beyond 2100 projections in an ocean acidification context; 2) the relatively benign environment in aquaculture (abundant food, disease protection, absence of predators) compared to the wild; 3) aquaculture species having been chosen due to their natural tolerance to the intensive conditions, including CO_2_ levels; or 4) the breeding of species within intensive aquaculture having further selected traits that confer tolerance to elevated CO_2_. We highlight this issue and outline the insights that climate change and aquaculture science can offer for both marine and freshwater settings. Integrating these two fields will stimulate discussion on the direction of future cross‐disciplinary research. In doing so, this article aimed to optimize future research efforts and elucidate effective mitigation strategies for managing the negative impacts of elevated CO_2_ on future aquatic ecosystems and the sustainability of fish and shellfish aquaculture.

## Introduction – Climate change, high CO_2_ and global food security

In 2015, atmospheric CO_2_ concentrations had risen to an annual average higher than 400 μatm the first time in over 800 000 years (Lüthi *et al*., 2008; Dlugokencky & Pieter, 2016), as a result of anthropogenic CO_2_ emissions. The potential implications of this postindustrial rise in CO_2_ were predicted over 110 years ago (Krogh, 1904); yet, it was only recently that governments agreed to take action on this issue. Despite 196 nations taking an unprecedented stance on climate change last year by signing the COP21 agreement to curtail emissions, CO_2_ concentrations are still projected to approach 1000 μatm by 2100 (Pörtner *et al*., 2014). Around a quarter of anthropogenic CO_2_ emissions have been absorbed by the oceans (Pörtner *et al*., 2014). Whilst this results in a phenomenon commonly referred to as ocean acidification, elevated atmospheric CO_2_ is also driving a large elevation in the average aquatic CO_2_ in fresh and brackish water systems, regardless of diurnal and seasonal variation. What is more, seasonal oscillations of aquatic CO_2_ in the future are predicted to amplify over time which will likely result in CO_2_ levels that exceed 1000 μatm for several months each year well before 2100 (McNeil & Sasse, 2016). Occurring simultaneously with warming, pollution, habitat degradation, disease outbreaks and overfishing, this aquatic acidification is therefore threatening not only aquatic ecosystems but also global food security (FAO, 2014, Porter *et al*., 2014).

Anthropogenic CO_2_ emissions accelerate alongside growth of the global human population, which is projected to exceed 9.6 billion by 2100 (Gerland *et al*., 2014). This same growth has also resulted in at least 80% of world fish stocks being overexploited (FAO, 2014, Pauly & Zeller, 2016). Aquaculture is therefore crucial to ensure the continued provision of fish and shellfish protein for human consumption, particularly for developing countries and small island nations (Bennett *et al*., 2016). Indeed, aquaculture is one of the fastest growing food‐producing industries globally (8.8% annual growth for the last 30 years) (FAO, 2014), and it is the only foreseeable way of increasing seafood^1^ production in the face of this human population expansion. However, to ensure aquaculture is able to maximize its potential for addressing global food security, a number of challenges need to be resolved concerning water availability and quality, environmental impacts and vulnerability to changing climatic conditions. Recirculating aquaculture systems (RAS) address many of these issues (Martins *et al*., 2010) and enable the sustainable intensification of aquaculture. These systems significantly reduce water requirements, relocate production of aquatic organisms away from a natural environmental setting and minimize environmental impacts. They also enable a tighter control of pathogens and other environmental parameters, potentially improving animal welfare and biosecurity, but they create some additional problems, particularly associated with accumulation of CO_2_.

## A common problem, two perspectives

**Figure 1:**
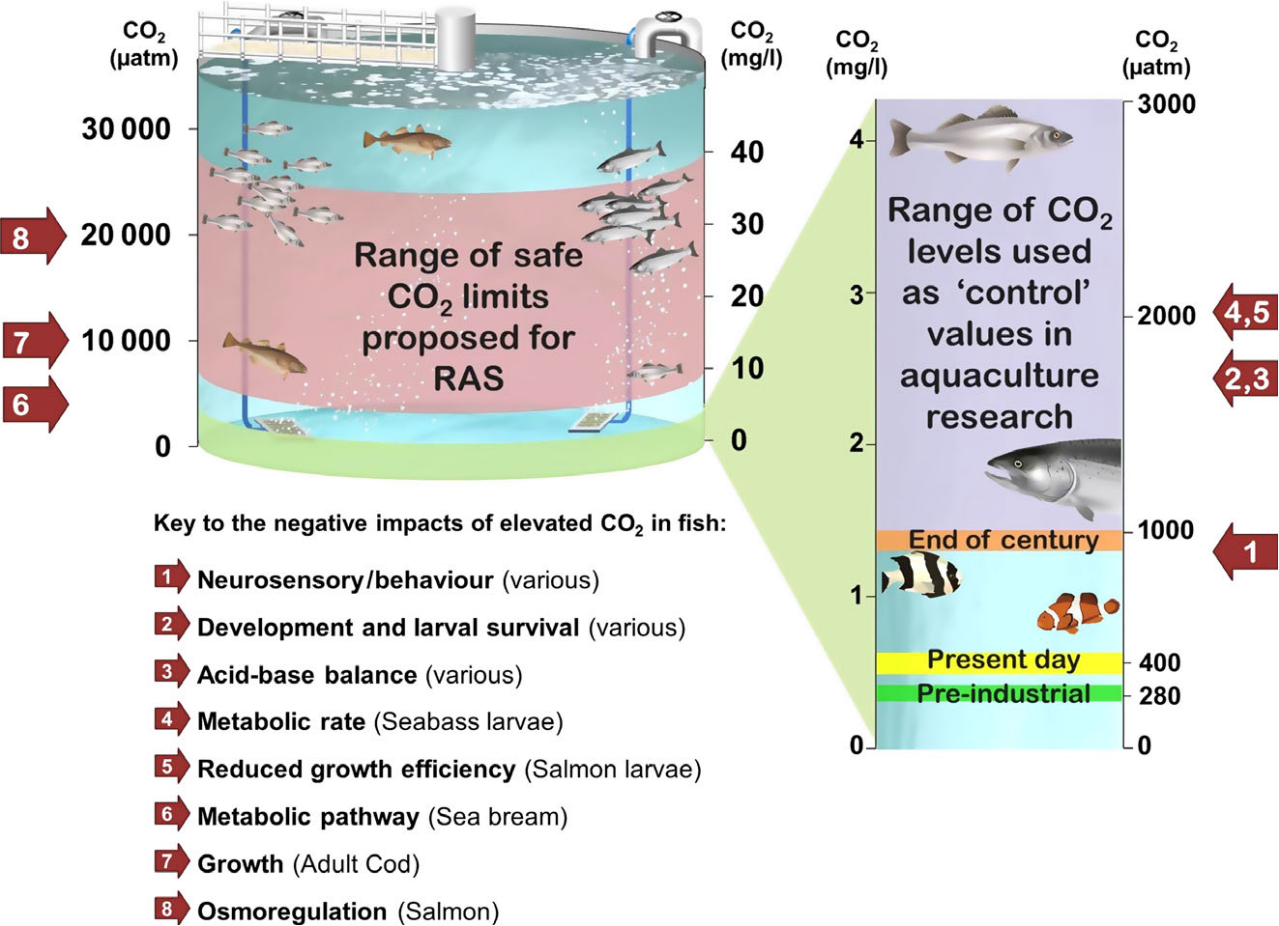
Diagrammatic representation of the levels at which elevated carbon dioxide is considered problematic within recirculating aquaculture systems (RAS) (caused by accumulation of excreted CO_2_ due to high stocking densities) and under global aquatic acidification (marine and freshwater, caused by rising atmospheric CO_2_). Numbered arrows, and corresponding key indicate the levels at which CO_2_ is demonstrated to have significant impacts on fish development, physiology and behaviour. The expanded view on the right side highlights CO_2_ levels in relation to climate change scenarios in greater detail (0–3000 μatm or 0–4 mg L^−1^). Conversion of CO_2_ levels between μatm and mg L^−1^ in this diagram is based on 35 psu sea water at 15°C. Fish images Kovalevska and Kazakov maksim/shutterstock.com. References corresponding to numbered arrows indicate levels of CO_2_ shown to have a significant impact of fish development, physiology or behaviour; 1) Hamilton *et al*. (2014), Jutfelt & Hedgärde (2013), Simpson *et al*. (2011), Nilsson *et al*. (2012); 2) Chambers *et al*. (2014), Frommel *et al*. (2012, 2014), Maneja *et al*. (2014), Tseng *et al*. (2013); 3) Esbaugh *et al*. (2016, 2012), Heuer *et al*. (2012); 4) Pope *et al*. (2014); 5) Ou *et al*. (2015); 6) Michaelidis *et al*. (2007); 7) Tirsgaard *et al*. (2015); & 8) Seidelin *et al*. (2001).

Physiologists have known for decades that raising the CO_2_ partial pressure in water to well above atmospheric levels (e.g. 10 000 μatm) has a direct effect on aquatic organisms in terms of acid–base and ion regulation, respiratory function and aerobic performance (Cameron & Randall, 1972). More recently, climate change studies have shown that CO_2_ levels projected for end of this century (e.g. 800–1000 μatm) can negatively affect development, physiology and fitness‐related behaviours in aquatic animals (see below). Due to the very high stocking densities achieved in most aquaculture settings, as well as the methods employed to control pH and O_2_, CO_2_ often accumulates, particularly in RAS. However, despite recent evidence on the potential detrimental effects of CO_2_ exposure at a level projected for 2100 (1000 μatm), the aquaculture industry was intensively farming fish and shellfish successfully at much higher CO_2_ levels long before the term ‘ocean acidification’ was coined. The levels at which the effects of CO_2_ are perceived as problematic, therefore, appear to differ greatly between the connected yet traditionally disparate fields of climate change and aquaculture (Fig. 1).

Current guidelines for intensive RAS propose safe CO_2_ levels ranging from 15 to 40 mg L^−1^ (Fivelstad *et al*., 1999, 2015; Blancheton, 2000; Petochi *et al*., 2011). These equate to an upper limit of CO_2_ ranging from >5000 to >30 000 μatm which are 12.5 to 75 times higher than current atmospheric levels, respectively. Furthermore, far from being an issue exclusively associated with RAS and finfish production, elevated CO_2_ levels appear synonymous with intensive aquaculture more generally. For example, over 40% of Norwegian salmon smolt hatcheries (flow‐through and RAS) report CO_2_ levels >5400 μatm (Noble *et al*., 2012), whereas Bangladeshi shrimp ponds are shown to experience CO_2_ levels averaging >17 000 μatm (Saksena *et al*., 2006; Sahu *et al*., 2013).

In stark contrast, recent studies emerging from aquatic acidification research have demonstrated that just 2.0‐ to 2.5‐fold increases in CO_2_ levels projected for the end of this century (e.g. 800–1000 μatm) can have dramatic and long‐lasting effects on the development, physiology and behaviour of both fish and invertebrates (Briffa *et al*., 2012; Schalkhausser *et al*., 2012; Heuer & Grosell, 2014; Watson *et al*., 2014; Welch *et al*., 2014). For example, exposure to 1000 μatm during early life cycle stages has been shown to result in reduced survival as well as a number of sublethal effects including tissue damage (e.g. Frommel *et al*., 2012, 2014; Chambers *et al*., 2014), altered calcification (e.g. Arnold *et al*., 2009; Maneja *et al*., 2013), reduced size (e.g. Talmage & Gobler, 2009; Maneja *et al*., 2014), reduced metabolic rate (e.g. Small *et al*., 2016), delayed development and altered gene expression (e.g. Tseng *et al*., 2013; Goncalves *et al*., 2016) in a range of different marine organisms. What is more, similar effects are also demonstrated in freshwater, with Ou *et al*. (2015) showing a significant effect of elevated CO_2_ (1000–2000 μatm) on the larval development of pink salmon *Oncorhynchus gorbuscha*. The authors reported a reduction in larval length, total wet and dry mass and reduced production efficiency (conversion of yolk into tissue growth).

In impacting a diverse array of aquatic organisms during early life stages, increased partial pressure of CO_2_ in aquatic environments above present‐day atmospheric levels is likely a bottleneck for organism production. This in turn would significantly impact aquaculture practices that depend upon a reliable source of larvae or juveniles. In 2007, these impacts were realized with the upwelling of elevated CO_2_, aragonite undersaturated sea water off the US west coast, significantly impacting oyster hatchery production as a direct result of changing climatic conditions (Barton *et al*., 2012). In addition to providing a case study in which to investigate the impact of ocean acidification on shellfish production globally, this event highlighted the significant advances achieved when climate change scientists and aquaculture practitioners work closely together. Unifying their research efforts to overcome this phenomenon, the climate change community and shellfish growers were able to successfully identify the root cause of this issue and put in place a number of mitigation strategies and monitoring protocols to minimize impacts in the future (Barton *et al*., 2015).

Far from being restricted to early life stages, a growing number of studies have also shown sublethal physiological impacts of elevated CO_2_ (range 1000–2000 μatm) in a number of species which include impacted respiratory gas transport, acid–base balance and gut carbonate excretion (e.g. Lannig *et al*., 2010; Esbaugh *et al*., 2012, 2016; Heuer *et al*., 2012; Wei *et al*., 2015). Rapid and efficient acid–base compensation has been demonstrated in a number of species at elevated CO_2_ concentrations (e.g. Melzner *et al*., 2009; Ern & Esbaugh, 2016; Lewis *et al*., 2016). However, such physiological responses incur energetic costs and could therefore have negative implications for production efficiency and body condition both in aquaculture and natural settings. Likewise, a wide range of behaviours are shown to be disrupted under elevated CO_2_, such as those linked to sensory stimuli (including smell, hearing and vision; e.g. Simpson *et al*., 2011; Nilsson *et al*., 2012; Roggatz *et al*., 2016) and cognitive‐related functions (such as lateralization, learning, bold‐shy phenotypes and escape behaviour; e.g. Schalkhausser *et al*., 2012; Jutfelt *et al*., 2013; Hamilton *et al*., 2014; Watson *et al*., 2014), which will have clear detrimental implications at the population level (Munday *et al*., 2009, 2010; Chivers *et al*., 2014). However, animals reared in many aquaculture settings are living in a relatively benign environment, being provided with abundant food, relatively constant environmental conditions, protection against disease and absence of a predation threat. Therefore, it is perhaps not surprising that the ecologically relevant physiological and behavioural disruptions caused by end‐of‐century CO_2_ levels in OA studies have not emerged from aquaculture studies. Equally it may be possible these behavioural effects have not been noted as they are not typically measured in aquaculture studies. Nevertheless, this does not mean that animals reared in an aquaculture setting are not facing problems associated with elevated CO_2_ that potentially influence their health and/or production efficiency.

## Cross‐discipline interaction to improve understanding of CO_2_ consequences

Given these contrasting views, combining the knowledge that has arisen from climate change and aquaculture research is crucial to allow a more in‐depth understanding of the physiological and ecological responses of aquatic animals to elevated CO_2_. The opportunity to compare these two fields directly is appealing, and should enable a more accurate prediction of the consequences of changing climatic conditions for wild populations and intensive aquaculture practices alike. However, at present, such comparison is not straightforward. This is partly due to the different experimental measures and reporting protocols typically adopted by each of these scientific fields. To facilitate this process, it would be fruitful to develop a collective research agenda and implement standard operating procedures with respect to hypothesis development, experimental outcomes and data reporting.

The comparison is also complicated by rather different species often being used in aquaculture compared to OA research, with the former inevitably relying on species that are amenable to domestication, which may go hand in hand with greater environmental tolerance. Indeed, when considering contrasting results from aquatic acidification and aquaculture fields, it is worth noting that responses from even closely related species can often vary significantly. For example, Ferrari *et al*. (2011) demonstrated a striking and unexpected difference for the impact of CO_2_ on the antipredator response of closely related damselfish species. Similarly, Lefevre (2016) and Heuer & Grosell (2014) highlight heterogeneity in physiological responses to elevated CO_2_ that argues against a unifying physiological theory for defining CO_2_ tolerance, and which needs to be accounted for when modelling and predicting the impacts of climate change. Indeed explaining such interspecies variability with respect to CO_2_ tolerance may provide a mechanistic understanding of why species used in aquaculture may be relatively tolerant to the CO_2_ levels prevalent within intensive production. However, it is important to note that even cod reared under end‐of‐century CO_2_ levels (1000 μatm) exhibit avoidance behaviour towards these conditions when presented with a choice, indicating negligible habituation and suggesting these conditions are unfavourable (Jutfelt & Hedgärde, 2013). Furthermore, a growing body of evidence shows that levels of CO_2_ experienced in aquaculture may be more detrimental than traditionally perceived (Heuer & Grosell, 2014). For example, Tirsgaard *et al*. (2015) and Ou *et al*. (2015) demonstrated detrimental effects of elevated CO_2_ in cod and salmon, respectively, species traditionally grown successfully under aquaculture settings. Exposure to 9200 μatm resulted in longer meal processing time and less efficient digestion in cod (Tirsgaard *et al*., 2015), whilst exposure to 2000 μatm reduced growth and production efficiency in salmon larvae (Ou *et al*., 2015), end‐point measures that are of specific importance to aquaculture production. Thus, differences between these two fields in the perceived impact of elevated CO_2_ cannot be explained solely by variability in interspecific responses. Measuring the impact of elevated CO_2_ on a diverse array of physiological and behavioural endpoints, not just those traditionally perceived as important for aquaculture production, is thus vital. It is also crucial to measure these responses in as many species as possible, both finfish and shellfish, as well as those traditionally perceived as CO_2_ tolerant and CO_2_ sensitive. By doing so, it will be possible to optimize water quality parameters within aquaculture, based on a species‐specific suite of physiological and behavioural CO_2_ tolerance endpoints. Targeting these conditions has the potential to maximize growth efficiency and health of aquaculture species, enhancing the sustainability of seafood production. With that aim, it is critical to understand the practical considerations of reducing and maintaining environmental conditions, particularly CO_2_, in an aquaculture context. Targets should thus be set that optimize productivity and welfare of the aquaculture species, but which are equally achievable in a practical and economical context (Noble *et al*., 2012).

**Figure 2:**
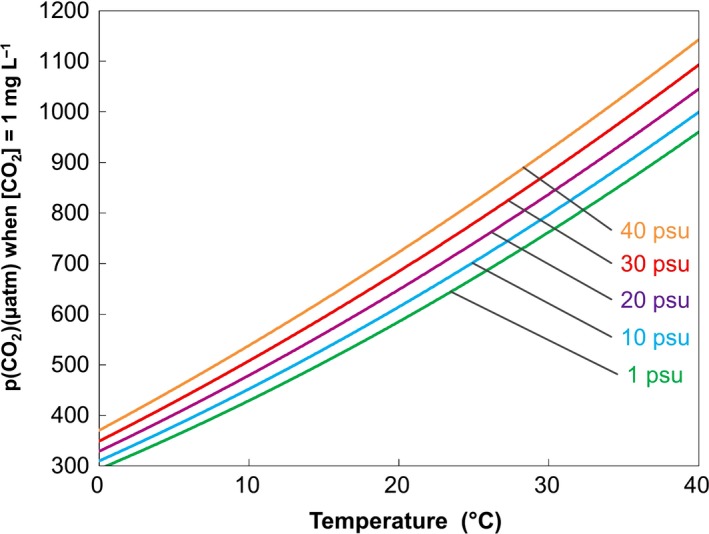
Schematic representation of the conversion of 1 mg L^−1^ dissolved CO_2_ concentration into partial pressure (μatm) at a range of different temperatures and salinities. This shows the very large influence of temperature in particular (up to 3.2‐fold higher partial pressure at the warmest temperature compared to the coolest) but also salinity (up to 26% higher partial pressure at the highest salinity compared to freshwater) on the CO_2_ partial pressure due to the impact these abiotic factors have on the solubility of CO_2_ in water (Dickson, 2011; Weiss, 1974). Conversion of dissolved CO_2_ in mg L^−1^ to partial pressure in μatm was undertaken using the CO2SYS programme (Pierrot *et al*., 2006), using dissociation constants from Mehrbach *et al*. (1973), refit by Dickson & Millero (1987), and KSO_4_ using Dickson (1990), with values for CO_2_ solubility at different temperatures and salinities checked against Weiss (1974).

To optimize research efforts and ensure data are both scientifically robust and comparable, a unified protocol for selecting, manipulating, measuring and finally reporting carbonate chemistry parameters is also needed. This is of particular importance given the methods of carbonate chemistry manipulation employed within intensive aquaculture, for example the addition of a strong alkali to buffer changes in pH, such as sodium hydroxide (NaOH), sodium bicarbonate (NaHCO_3_), calcium hydroxide (Ca(OH)_2_) or calcium oxide (CaO). This is the most commonly used of all water chemistry quality management practices in aquaculture, being typically employed in a diverse array of aquaculture settings (Boyd *et al*., 2016). However, this method of pH compensation additionally elevates alkalinity, often significantly beyond any natural analogue (Ellis *et al*., in preparation), and depending on the alkali used can have dramatic indirect effects on additional water chemistry parameters, some of which are shown themselves to influence a number physiological processes in aquatic organisms (Boyd *et al*., 2016; Middlemiss *et al*., 2016). A further crucial issue is the selection of experimental controls representing present‐day CO_2_ levels (400 μatm), and we propose this should be a common reference point for both climate change and aquaculture researchers. Control levels employed within aquaculture research typically exceed 1000 μatm (range 1000–3000 μatm) (Fivelstad *et al*., 1999; Petochi *et al*., 2011) and thus surpass most of the ‘high CO_2_’ treatments used as end‐of‐century projections in climate change studies. To complicate matters further, reporting CO_2_ levels as mg L^−1^ in aquaculture studies overlooks the impact of temperature and salinity on the solubility of CO_2_ and the resulting impact these have on the partial pressure of this gas (Weiss, 1974; Dickson, 2011). For example, for the same mg L^−1^ concentration, the actual partial pressure of CO_2_ varies by more than threefold between cold freshwater and warm sea water (Fig. 2). This is critical because it is partial pressure (not the mg L^−1^ concentration) that determines the internal (blood) levels of CO_2_ and its impact on physiology, behaviour, growth, etc. At present, the scarcity of sufficient water chemistry parameters being presented, the lack of environmentally relevant controls and the prevalence of reporting CO_2_ levels as mg L^−1^ in aquaculture literature preclude an unambiguous comparison between data from these two fields.

Finally, understanding and reporting the provenance of the study species/population will be important to enable a more in‐depth assessment of CO_2_ tolerance, that is whether animals are wild‐caught, laboratory‐bred or reared within an aquaculture setting (potentially already at very high CO_2_ when considered in a climate change experimental context). It is fair to say that many (though not all) laboratory‐based climate change studies benefit from easy access to study species available from aquaculture. The systematic selection of traits of interest by the aquaculture industry, such as fast growth and resistance to pathogens, has inherently selected for good performance under intensive farming conditions. In that context, it is possible, and even likely, that additional nontarget traits have also been selected, potentially including those involved in CO_2_ tolerance. Indeed, enhanced CO_2_ tolerance has been demonstrated in selectively bred populations of the Sydney rock oyster, compared to its wild‐type congeners (Parker *et al*., 2011, 2015; Thompson *et al*., 2015). Furthermore, as demonstrated by Malvezzi *et al*. (2015), early life survival at elevated CO_2_ concentrations can have a significant additive genetic element (i.e. highly heritable), which under sufficient selection pressure could elicit a strong and rapid evolutionary response. It is highly likely therefore that aquaculture practices operating at elevated CO_2_ concentrations would elicit sufficient selection pressure to directly select for CO_2_ tolerance during early life stages, leading to the rapid evolution of the population in just a few generations. Thus, exploring the traits selected for in broodstock within intensive aquaculture offers a fascinating opportunity to investigate multigenerational adaptation to CO_2_ levels experienced under intensive production conditions in aquaculture species. In addition, it will be vital to undertake multigenerational studies in order to discern the transgenerational acclimation to elevated CO_2_ of different fish species with respect to different behavioural (e.g. Welch *et al*., 2014) and physiological (e.g. Miller *et al*., 2012) endpoints. Combining the understanding from these two fields will therefore help determine the physiological basis for CO_2_ tolerance, determine its true ecological consequence and determine its ecological impacts over relevant timescales.

## Conclusions

The yield from wild capture fisheries has plateaud since the late 1980s and human consumption from aquaculture exceeded that from wild sources for the first time in 2014 (FAO, 2014). Furthermore, as stated previously, aquaculture is likely to be the only pathway for increasing seafood production in the future. Moving from a capture to a culture mentality requires a shift in attitude that will require time, a luxury that is ill‐afforded in the rapidly changing environment of the Anthropocene. Creating opportunities for the aquatic acidification community and the aquaculture industry to work together should help to speed up this process and enable the aquaculture industry to rapidly adapt by using better‐informed decisions to a) optimize the water chemistry conditions within intensive aquaculture to suit the species and/or b) select traits within the species to suit intensive aquaculture conditions. This will help address the environmental, economic and social impacts of this developing sector towards a sustainable intensification of production, enhancing food security and its resilience to climate change. Equally, this cross‐discipline interaction should also improve our capability to predict and mitigate the consequences of the changing chemistry for natural ecosystems in a future ‘high’ CO_2_ world.

## Author contributions

R.W.W. won the funding for aquaculture and aquatic acidification projects that stimulated this article and produced Fig. 2. R.E led the formulation of the paper and produced Fig. 1. M.U compiled the initial draft. All authors contributed equally to discussions, figure development, editing and production of the final manuscript.

## Competing financial interests

The authors declare no competing financial interests.

## Note

1 Seafood in this context refers to all fish and shellfish species produced under fresh, brackish or marine conditions and intended for human consumption

## References

[gcb13515-bib-0001] Arnold KE , Findlay HS , Spicer JI , Daniels CL , Boothroyd D (2009) Effect of CO_2_‐related acidification on aspects of the larval development of the European lobster, *Homarus gammarus* (L.). Biogeosciences, 6, 1747–1754.

[gcb13515-bib-0002] Barton A , Hales B , Waldbusser GG , Langdon C , Feely RA (2012) The Pacific oyster, Crassostrea gigas, shows negative correlation to naturally elevated carbon dioxide levels: implications for near‐term ocean acidification effects. Limnology and Oceanography, 57, 698–710.

[gcb13515-bib-0003] Barton A , Waldbusser GG , Feely RA , Hales B , Langdon CJ (2015) Impacts of coastal acidification on the Pacific Northwest shellfish industry and adaptation strategies implemented in response. Oceanography, 28, 146–159.

[gcb13515-bib-0004] Bennett NJ , Blythe J , Tyler S , Ban NC (2016) Communities and change in the anthropocene: understanding social‐ecological vulnerability and planning adaptations to multiple interacting exposures. Regional Environmental Change, 16, 907–926.

[gcb13515-bib-0005] Blancheton JP (2000) Developments in recirculation systems for Mediterranean fish species. Aquacultural Engineering, 22, 17–31.

[gcb13515-bib-0006] Boyd CE , Tucker CS , Somridhivej B (2016) Alkalinity and hardness: critical but elusive concepts in aquaculture. Journal of the World Aquaculture Society, 47, 6–41.

[gcb13515-bib-0007] Briffa M , de la Haye K , Munday PL (2012) High CO_2_ and marine animal behaviour: potential mechanisms and ecological consequences. Marine Pollution Bulletin, 64, 1519–1528. 22749063 10.1016/j.marpolbul.2012.05.032

[gcb13515-bib-0008] Cameron JN , Randall DJ (1972) The effect of increased ambient CO_2_ on arterial CO_2_ tension, CO_2_ content and pH in rainbow trout. Journal of Experimental Biology, 57, 673–680. 4651664 10.1242/jeb.57.3.673

[gcb13515-bib-0009] Chambers RC , Candelmo AC , Habeck EA *et al* (2014) Effects of elevated CO_2_ in the early life stages of summer flounder, *Paralichthys dentatus*, and potential consequences of ocean acidification. Biogeosciences, 11, 1613–1626.

[gcb13515-bib-0010] Chivers DP , McCormick MI , Nilsson GE *et al* (2014) Impaired learning of predators and lower prey survival under elevated CO_2_: a consequence of neurotransmitter interference. Global Change Biology, 20, 515–522. 23765546 10.1111/gcb.12291

[gcb13515-bib-0011] Dickson AG (1990) Standard potential of the reaction: and the standard acidity constant of the ion HSO_4_ ^‐^ in synthetic sea water from 273.15 to 318.15 K. The Journal of Chemical Thermodynamics, 22, 113–127.

[gcb13515-bib-0012] Dickson AG (2011) The carbon dioxide system in seawater: equilibrium chemistry and measurements In: Guide to Best Practices for Ocean Acidification Research and Data Reporting. (eds Riebesell U, Fabry VJ, Hansson L, Gattuso JP), pp 17–40. Publications Office of the European Union, Luxembourg.

[gcb13515-bib-0013] Dickson AG , Millero FJ (1987) A comparison of the equilibrium constants for the dissociation of carbonic acid in seawater media. Deep‐Sea Research, 34, 1733–1743.

[gcb13515-bib-0014] Dlugokencky E , Pieter T (2016) Trends in Atmospheric Carbon Dioxide. (ed Division N‐E‐GM ).

[gcb13515-bib-0015] Ern R , Esbaugh AJ (2016) Hyperventilation and blood acid–base balance in hypercapnia exposed red drum (*Sciaenops ocellatus*). Journal of Comparative Physiology B, 186, 447–460. 10.1007/s00360-016-0971-7 26922790

[gcb13515-bib-0016] Esbaugh AJ , Heuer R , Grosell M (2012) Impacts of ocean acidification on respiratory gas exchange and acid–base balance in a marine teleost, *Opsanus beta* . Journal of Comparative Physiology B, 182, 921–934. 10.1007/s00360-012-0668-5 22581071

[gcb13515-bib-0017] Esbaugh AJ , Ern R , Nordi WM , Johnson AS (2016) Respiratory plasticity is insufficient to alleviate blood acid–base disturbances after acclimation to ocean acidification in the estuarine red drum, *Sciaenops ocellatus* . Journal of Comparative Physiology B, 186, 97–109. 10.1007/s00360-015-0940-6 26487347

[gcb13515-bib-0018] FAO (2014) The State of World Fisheries and Aquaculture ‐ Opportunities and Challenges. Food and Agriculture Organization of the United Nations, Rome, Italy.

[gcb13515-bib-0019] Ferrari MCO , Dixson DL , Munday PL , McCormick MI , Meekan MG , Sih A , Chivers DP (2011) Intrageneric variation in antipredator responses of coral reef fishes affected by ocean acidification: implications for climate change projections on marine communities. Global Change Biology, 17, 2980–2986.

[gcb13515-bib-0020] Fivelstad S , Olsen AB , Kloften H , Ski H , Stefansson S (1999) Effects of carbon dioxide on Atlantic salmon (*Salmo salar*) smolts at constant pH in bicarbonate rich freshwater. Aquaculture, 178, 171–187.

[gcb13515-bib-0021] Fivelstad S , Kvamme K , Handeland S , Fivelstad M , Olsen AB , Hosfeld CD (2015) Growth and physiological models for Atlantic salmon (*Salmo salar L*.) parr exposed to elevated carbon dioxide concentrations at high temperature. Aquaculture, 436, 90–94.

[gcb13515-bib-0022] Frommel AY , Maneja R , Lowe D *et al* (2012) Severe tissue damage in Atlantic cod larvae under increasing ocean acidification. Nature Climate Change, 2, 42–46.

[gcb13515-bib-0023] Frommel AY , Maneja R , Lowe D *et al* (2014) Organ damage in Atlantic herring larvae as a result of ocean acidification. Ecological Applications, 24, 1131–1143. 25154101 10.1890/13-0297.1

[gcb13515-bib-0024] Gerland P , Raftery AE , Ševčíková H *et al* (2014) World population stabilization unlikely this century. Science, 348, 234–237. 10.1126/science.1257469 PMC4230924 25301627

[gcb13515-bib-0025] Goncalves P , Anderson K , Thompson EL , Melwani A , Parker L , Ross PM , Raftos DA (2016) Rapid transcriptional acclimation following transgenerational exposure of oysters to ocean acidification. Molecular Ecology, 25, 4836–4849. 27543886 10.1111/mec.13808

[gcb13515-bib-0026] Hamilton TJ , Holcombe A , Tresguerres M (2014) CO_2_‐induced ocean acidification increases anxiety in rockfish via alteration of GABAA receptor functioning. Proceedings of the Royal Society of London B: Biological Sciences, 281, 20132509. 10.1098/rspb.2013.2509 PMC3866405 24285203

[gcb13515-bib-0027] Heuer RM , Grosell M (2014) Physiological impacts of elevated carbon dioxide and ocean acidification on fish. American Journal of Physiology‐Regulatory, Integrative and Comparative Physiology, 307, R1061–R1084. 10.1152/ajpregu.00064.2014 25163920

[gcb13515-bib-0028] Heuer RM , Esbaugh AJ , Grosell M (2012) Ocean acidification leads to counterproductive intestinal base loss in the Gulf Toadfish (*Opsanus beta*). Physiological and Biochemical Zoology: Ecological and Evolutionary Approaches, 85, 450–459. 10.1086/667617 22902373

[gcb13515-bib-0029] Jutfelt F , Hedgärde M (2013) Atlantic cod actively avoid CO_2_ and predator odour, even after long‐term CO_2_ exposure. Frontiers in Zoology, 10, 1–8. 24373523 10.1186/1742-9994-10-81 PMC3880174

[gcb13515-bib-0030] Jutfelt F , Bresolin de Souza K , Vuylsteke A , Sturve J (2013) Behavioural disturbances in a temperate fish exposed to sustained high‐CO_2_ levels. PLoS ONE, 8, e65825. 23750274 10.1371/journal.pone.0065825 PMC3672104

[gcb13515-bib-0031] Krogh A (1904) On the tension of carbonic acid in natural waters and especially in the sea. Meddelelser om Grønland, 26, 231–342.

[gcb13515-bib-0032] Lannig G , Eilers S , Pörtner HO , Sokolva IM , Bock C (2010) Impact of ocean acidification on energy metabolism of oyster, *Crassostrea gigas* ‐ changes in metabolic pathways and thermal response. Marine Drugs, 8, 2318–2339. 20948910 10.3390/md8082318 PMC2953406

[gcb13515-bib-0033] Lefevre S (2016) Are global warming and ocean acidification conspiring against marine ectotherms? A meta‐analysis of the respiratory effects of elevated temperature, high CO_2_ and their interaction Conservation Physiology, 4, cow009. 27382472 10.1093/conphys/cow009 PMC4922249

[gcb13515-bib-0034] Lewis C , Ellis RP , Vernon E , Elliot K , Newbatt S , Wilson RW (2016) Ocean acidification increases copper toxicity differentially in two key marine invertebrates with distinct acid‐base responses. Scientific Reports, 6, 21554. 26899803 10.1038/srep21554 PMC4761931

[gcb13515-bib-0035] Lüthi D , Le Floch M , Bereiter B *et al* (2008) High‐resolution carbon dioxide concentration record 650,000‐800,000 years before present. Nature, 453, 379–382. 18480821 10.1038/nature06949

[gcb13515-bib-0036] Malvezzi AJ , Murray CS , Feldheim KA *et al* (2015) A quantitative genetic approach to assess the evolutionary potential of a coastal marine fish to ocean acidification. Evolutionary Applications, 8, 352–362. 25926880 10.1111/eva.12248 PMC4408146

[gcb13515-bib-0037] Maneja R , Frommel A , Geffen A , Folkvord A , Piatkowski U , Chang M , Clemmesen C (2013) Effects of ocean acidification on the calcification of otoliths of larval Atlantic cod *Gadus morhua* . Marine Ecology Progress Series, 477, 251–258.

[gcb13515-bib-0038] Maneja RH , Dineshram R , Thiyagarajan V *et al* (2014) The proteome of Atlantic herring (*Clupea harengus* L.) larvae is resistant to elevated pCO_2_ . Marine Pollution Bulletin, 86, 154–160. 25110053 10.1016/j.marpolbul.2014.07.030

[gcb13515-bib-0039] Martins CIM , Eding EH , Verdegem MCJ *et al* (2010) New developments in recirculating aquaculture systems in Europe: a perspective on environmental sustainability. Aquacultural Engineering, 43, 83–93.

[gcb13515-bib-0040] McNeil BI , Sasse TP (2016) Future ocean hypercapnia driven by anthropogenic amplification of the natural CO_2_ cycle. Nature, 529, 383–386. 26791726 10.1038/nature16156

[gcb13515-bib-0041] Mehrbach C , Culberson CH , Hawley JE , Pytkowicz RM (1973) Measurements of the apparent dissociation constants of carbonic acid in seawater at atmospheric pressure. Limnology and Oceanography, 18, 897–906.

[gcb13515-bib-0042] Melzner F , Göbel S , Langenbuch M , Gutowska MA , Pörtner H‐O , Lucassen M (2009) Swimming performance in Atlantic Cod (*Gadus morhua*) following long‐term (4–12 months) acclimation to elevated seawater. Aquatic Toxicology, 92, 30–37. 19223084 10.1016/j.aquatox.2008.12.011

[gcb13515-bib-0043] Michaelidis B , Spring A , Pörtner HO (2007) Effects of long‐term acclimation to environmental hypercapnia on extracellular acid–base status and metabolic capacity in Mediterranean fish *Sparus aurata* . Marine Biology, 150, 1417–1429.

[gcb13515-bib-0044] Middlemiss KL , Urbina MA , Wilson RW (2016) Effects of seawater alkalinity on calcium and acid–base regulation in juvenile European lobster (*Homarus gammarus*) during a moult cycle. Comparative Biochemistry and Physiology Part A: Molecular & Integrative Physiology, 193, 22–28. 10.1016/j.cbpa.2015.12.002 26691956

[gcb13515-bib-0045] Miller GM , Watson S‐A , Donelson JM , McCormick MI , Munday PL (2012) Parental environment mediates impacts of increased carbon dioxide on a coral reef fish. Nature Climate Change, 2, 858–861.

[gcb13515-bib-0046] Munday PM , Dixson DL , Donelson JM , Jones GP , Pratchett MS , Devitsina GV , Døving KB (2009) Ocean acidification impairs olfactory discrimination and homing ability of a marine fish. Proceedings of the National Academy of Science USA, 106, 1848–1852. 10.1073/pnas.0809996106 PMC2644126 19188596

[gcb13515-bib-0047] Munday PL , Dixson DL , McCormick MI , Meekan M , Ferrari MCO , Chivers DP (2010) Replenishment of fish populations is threatened by ocean acidification. Proceedings of the National Academy of Science USA, 107, 12930–12934. 10.1073/pnas.1004519107 PMC2919925 20615968

[gcb13515-bib-0048] Nilsson GE , Dixson DL , Domenici P , McCormick MI , Sørensen C , Watson S‐A , Munday PL (2012) Near‐future carbon dioxide levels alter fish behaviour by interfering with neurotransmitter function. Nature Climate Change, 2, 201–204.

[gcb13515-bib-0049] Noble C , Kankainen M , Setälä J , Berrill IK , Ruohonen K , Damsgård B , Toften H (2012) The bio‐economic costs and benefits of improving productivity and fish welfare in aquaculture: utilizing CO_2_ stripping technology in norwegian atlantic salmon smolt production. Aquaculture Economics & Management, 16, 414–428.

[gcb13515-bib-0050] Ou M , Hamilton TJ , Eom J *et al* (2015) Responses of pink salmon to CO_2_‐induced aquatic acidification. Nature Climate Change, 5, 950–955.

[gcb13515-bib-0051] Parker L , Ross PM , O'Connor WA (2011) Populations of the Sydney rock oyster, *Saccostrea glomerata*, vary in response to ocean acidification. Marine Biology, 158, 689–697.

[gcb13515-bib-0052] Parker LM , O'Connor WA , Raftos DA , Pörtner H‐O , Ross PM (2015) Persistence of positive carryover effects in the oyster, *Saccostrea glomerata,* following transgenerational exposure to ocean acidification. PLoS ONE, 10, e0132276. 26147612 10.1371/journal.pone.0132276 PMC4493068

[gcb13515-bib-0053] Pauly D , Zeller D (2016) Catch reconstructions reveal that global marine fisheries catches are higher than reported and declining. Nature Communications, 7, 10244. 10.1038/ncomms10244 PMC4735634 26784963

[gcb13515-bib-0054] Petochi T , Di Marco P , Priori A , Finoia MG , Mercatali I , Marino G (2011) Coping strategy and stress response of European sea bass *Dicentrarchus labrax* to acute and chronic environmental hypercapnia under hyperoxic conditions. Aquaculture, 315, 312–320.

[gcb13515-bib-0055] Pierrot EC , Lewis E , Wallace DWR (2006) CO_2_SYS Dos Program Developed for CO_2_ System Calculations. ORNL/CDIAC‐105. Carbon Dioxide Information Analysis Centre, Oak Ridge National Laboratory, US Department of Energy, Oak Ridge, TN.

[gcb13515-bib-0056] Pope EC , Ellis RP , Scolamacchia M *et al* (2014) European sea bass, *Dicentrarchus labrax,* in a changing ocean. Biogeosciences, 11, 2519–2530.

[gcb13515-bib-0057] Porter JR , Xie L , Challinor AJ *et al* (2014) Food security and food production systems In: Climate Change 2014: Impacts, Adaptation, and Vulnerability. Part A: Global and Sectoral Aspects. Contribution of Working Group II to the Fifth Assessment Report of the Intergovernmental Panel on Climate Change. (eds Field CB, Barros VR, Dokken DJ, Mach KJ, Mastrandrea MD, Bilir TE, Chatterjee M, Ebi KL, Estrada YO, Genova RC, Girma B, Kissel ES, Levy AN, Maccracken S, Mastrandrea PR, White LL), pp. 485–534. Cambridge University Press, Cambridge, United Kingdom and New York, NY, USA.

[gcb13515-bib-0058] Pörtner HO , Karl DM , Boyd PW *et al* (2014) Ocean systems In: Climate Change 2014: Impacts, Adaptation, and Vulnerability. Part A: Global and Sectoral Aspects. Contribution of Working Group II to the Fifth Assessment Report of the Intergovernmental Panel on Climate Change. (eds Field CB, Barros VR, Dokken DJ, Mach KJ, Mastrandrea MD, Bilir TE, Chatterjee M, Ebi KL, Estrada YO, Genova RC, Girma B, Kissel ES, Levy AN, Maccracken S, Mastrandrea PR, White LL), pp. 411–484. Cambridge University Press, Cambridge, United Kingdom and New York, NY, USA.

[gcb13515-bib-0059] Roggatz CC , Lorch M , Hardege JD , Benoit DM (2016) Ocean acidification affects marine chemical communication by changing structure and function of peptide signalling molecules. Global Change Biology, 22, 3914–3926. 27353732 10.1111/gcb.13354

[gcb13515-bib-0060] Sahu BC , Adhikari S , Mahapatra AS , Dey L (2013) Carbon, nitrogen, and posphorous budget in scampi (*Macrobrachium rosenbergii*) culture ponds. Environmental Monitoring and Assessment, 185, 10157–10166. 23832231 10.1007/s10661-013-3320-2

[gcb13515-bib-0061] Saksena DN , Gaidhane DM , Singh H (2006) Limnology of Kharland (saline) ponds of Ratnagiri, Maharashtra in relation to prawn culture potential. Journal of Environmental Biology, 27, 49–53. 16850875

[gcb13515-bib-0062] Schalkhausser B , Bock C , Stemmer K , Brey T , Pörtner H‐O , Lannig G (2012) Impact of ocean acidification on escape performance of the king scallop, *Pecten maximus*, from Norway. Marine Biology, 160, 1995–2006.

[gcb13515-bib-0063] Seidelin M , Brauner CJ , Jensen FB , Madsen SS (2001) Vacuolar‐Type H^+^‐ATPase and Na^+^, K^+^‐ATPase expression in gills of Atlantic Salmon (*Salmo salar*) during isolated and combined exposure to hyperoxia and hypercapnia in fresh water. Zoological Science, 18, 1199–1205. 11911075 10.2108/zsj.18.1199

[gcb13515-bib-0064] Simpson SD , Munday PL , Wittenrich ML , Manassa R , Dixson DL , Gagliano M , Yan HY (2011) Ocean acidification erodes crucial auditory behaviour in a marine fish. Biology Letters, 7, 917–920. 21632617 10.1098/rsbl.2011.0293 PMC3210647

[gcb13515-bib-0065] Small DP , Calosi P , Boothroyd D , Widdicombe S , Spicer JI (2016) The sensitivity of the early benthic juvenile stage of the European lobster *Homarus gammarus* (L.) to elevated pCO_2_ and temperature. Marine Biology, 163, 1–12.

[gcb13515-bib-0066] Talmage SC , Gobler CJ (2009) The effects of elevated carbon dioxide concentrations on the metamorphosis, size, and survival of larval hard clams (*Mercenaria mercenaria*), bay scallops (*Argopecten irradians*), and Eastern oysters (*Crassostrea virginica*). Limnology and Oceanography, 54, 2072–2080.

[gcb13515-bib-0067] Thompson EL , O'Connor W , Parker L , Ross P , Raftos DA (2015) Differential proteomic responses of selectively bred and wild‐type Sydney rock oyster populations exposed to elevated CO_2_ . Molecular Ecology, 24, 1248–1262. 25689603 10.1111/mec.13111

[gcb13515-bib-0068] Tirsgaard B , Moran D , Steffensen JF (2015) Prolonged SDA and reduced digestive efficiency under elevated CO_2_ may explain reduced growth in Atlantic cod (*Gadus morhua*). Aquatic Toxicology, 158, 171–180. 25438123 10.1016/j.aquatox.2014.11.009

[gcb13515-bib-0069] Tseng Y‐C , Hu MY , Stumpp M , Lin L‐Y , Melzner F , Hwang P‐P (2013) CO_2_‐driven seawater acidification differentially affects development and molecular plasticity along life history of fish (*Oryzias latipes*). Comparative Biochemistry and Physiology Part A: Molecular & Integrative Physiology, 165, 119–130. 10.1016/j.cbpa.2013.02.005 23416137

[gcb13515-bib-0070] Watson S‐A , Lefevre S , McCormick MI , Domenici P , Nilsson GE , Munday PL (2014) Marine mollusc predator‐escape behaviour altered by near‐future carbon dioxide levels. Proceedings of the National Academy of Sciences B, 281, 20132377. 10.1098/rspb.2013.2377 PMC3843832 24225456

[gcb13515-bib-0071] Wei L , Wang Q , Ning X *et al* (2015) Combined metabolome and proteome analysis of the mantle tissue from Pacific oyster *Crassostrea gigas* exposed to elevated pCO_2_ . Comparative Biochemistry and Physiology Part D: Genomics and Proteomics, 13, 16–23. 10.1016/j.cbd.2014.12.001 25559488

[gcb13515-bib-0072] Weiss RF (1974) Carbon dioxide in water and seawater: the solubility of a non‐ideal gas. Marine Chemistry, 2, 203–215.

[gcb13515-bib-0073] Welch MJ , Watson S‐A , Welsh JQ , McCormick MI , Munday PL (2014) Effects of elevated CO_2_ on fish behaviour undiminished by transgenerational acclimation. Nature Climate Change, 4, 1086–1089.

